# Evolutionary trade-off between innate and acquired immune defences in birds

**DOI:** 10.1186/s12983-023-00511-1

**Published:** 2023-09-08

**Authors:** Piotr Minias, Wei-Xuan V. -H. Peng, Kevin D. Matson

**Affiliations:** 1 Department of Biodiversity Studies and Bioeducation, Faculty of Biology and Environmental Protection, University of Łódź, Banacha 1/3, 90-237 Łódź, Poland; 2 Wildlife Ecology and Conservation Group, Wageningen University & Research, Droevendaalsesteeg 3a, 6708PB Wageningen, Netherlands

**Keywords:** Birds, Copy number variation, Evolutionary trade-off, Haemagglutination-haemolysis assay, Major histocompatibility complex

## Abstract

**Background:**

The development, maintenance, and use of immune defences are costly. Therefore, animals face trade-offs in terms of resource allocation within their immune system and between their immune system and other physiological processes. To maximize fitness, evolution may favour investment in one immunological defence or subsystem over another in a way that matches a species broader life history strategy. Here, we used phylogenetically-informed comparative analyses to test for relationships between two immunological components. Natural antibodies and complement were used as proxies for the innate branch; structural complexity of the major histocompatibility complex (MHC) region was used for the acquired branch.

**Results:**

We found a negative association between the levels of natural antibodies (i.e., haemagglutination titre) and the total MHC gene copy number across the avian phylogeny, both at the species and family level. The family-level analysis indicated that this association was apparent for both MHC-I and MHC-II, when copy numbers within these two MHC regions were analysed separately. The association remained significant after controlling for basic life history components and for ecological traits commonly linked to pathogen exposure.

**Conclusion:**

Our results provide the first phylogenetically robust evidence for an evolutionary trade-off within the avian immune system, with a more developed acquired immune system (i.e., more complex MHC architecture) in more derived bird lineages (e.g., passerines) being accompanied by an apparent downregulation of the innate immune system.

**Supplementary Information:**

The online version contains supplementary material available at 10.1186/s12983-023-00511-1.

## Background

All metazoans experience diverse encounters with microorganisms, multicellular parasites, and self and non-self molecules, all of which shape their immunological strategies. While invertebrates primarily rely on innate (or non-specific) immune defences [1] (but see [2] for evidence of immunological specificity in invertebrates), the evolution of vertebrates has favoured the emergence of acquired (or adaptive) immune defences, which tend to target threats, particularly repeated ones, with greater specificity. To optimally defend themselves, vertebrates seemingly rely on different mixes of defences rooted in both the innate and adaptive subsystems, which are functionally intertwined [3]. For example, in the context of limiting infection by pathogenic microorganisms, the innate immune system has evolved to detect invariant non-self features of microorganisms (e.g., pathogen-associated molecular patterns, PAMPs) via pattern-recognition receptors (PRRs). PRRs can stimulate innate defences (e.g., serum lectin PRRs can activate the complement system and collaborate with natural antibodies [3, 4]) and acquired defences (including both B and T cell responses [4]). In the latter case, microbial antigens are presented to T cells by the major histocompatibility complex (MHC) molecules, which are central in vertebrate acquired immunity.

Despite tight mechanistic interrelationships between the innate and acquired branches of the vertebrate immune system, coevolution of the two may be shaped by trade-offs. In general, the development, maintenance, and use of the immune system are costly in terms of energy and biochemical substrates. If not for the requirements of the immune system, resources could be allocated to other physiological processes, including growth and reproduction [5, 6]. Thus, an overall immunological strategy (i.e., mix of defences) should balance the benefits of defence against its physiological costs (which could be in terms of energy, autoimmunity, or ultimately fitness) [7]. However, differences in cost–benefit ratios among defences can also lead to trade-offs within the immune system. For instance, acquired defences seem to incur lower costs in terms of required resources and collateral (autoimmune) damage compared to innate defences. The latter can necessitate the elevated levels of protein production, for example during an acute phase response, and can be self-reactive due to their inherent lack of specificity [7–9]. Thus, trade-offs within the immune system may not only depend on the allocation of energy or other limited resources, but also on fitness consequences stemming from other physiological impacts. For example, independent of energy, one highly effective immune defence could relax the need for a different defence that is linked to great risk of collateral damage and larger reductions in fitness. Furthermore, the functional profiles of innate and acquired defences also differ: innate defences can more quickly combat novel threats; acquired defences can better protect against repeated exposures, for example via immunological memory [7]. Thus, optimization of immunological strategy may be governed by the ecology and life history of a species [10]. Slow-living animal species with relatively long lifespans may invest more in acquired defences than fast-living ones, which may have lifespans so short that secondary encounters with pathogens are rare (pace-of-life hypothesis [9, 11]). Alternatively, pathogen pressure may be a key determinant of species immunological strategy (antigen exposure hypothesis [12]). Species from pathogen-rich environments may either invest more in their immune system overall or prioritize investment in low-cost acquired immunity when resources are limited [13]. Although the theoretical framework for trade-offs in the evolution of innate and acquired defences is well developed [7], comparative support is scant [14, 15]. While there is accumulating evidence for trade-offs between innate and acquired immunity at the individual level [16, 17], it remains unknown if phylogenetic- and individual-scale patterns match, indicating a scale independency in immunological adaptations [18].

Here, we used phylogenetically-informed comparative analyses to test for relationships between innate and acquired immune defences. We used haemagglutination and haemolysis titres as indices of the innate branch of the immune system [19]. Higher titres of haemagglutination and haemolysis reflect higher plasma concentrations of natural antibodies and complement, respectively [19]. Natural antibodies are germline encoded antibodies that are produced constitutively and without prior exposure to the wide range of antigens they recognize [4, 20]. The complement system (henceforth referred to as complement) is a proinflammatory protein network that results in the lysis of foreign cells [21]. These two defences are methodologically repeatable [22] and seemingly represent species traits (see below). Both natural antibodies and complement are relatively unaffected by prior antigenic exposure or immune responses, in contrast with specific antibody titres (which spike following antigen exposure) or acute phase protein concentrations (which can change by orders of magnitude during an inflammatory response) [22]. Furthermore, both measures are expected to be present relatively consistently throughout the lifetime of an individual, i.e., do not show age-related declines [23, 24] or strong stochastic variation in time, thus yielding relatively high temporal repeatability within individuals [25]. Consequently, haemagglutination and haemolysis titres are thought to characterize among-species variation in innate immune function (being repeatable at the inter-specific level, as shown in the Methods section below), and they have been used for this purpose in broad-scale comparative analyses across avian phylogeny [9, 25, 26]. While natural antibodies and complement are quintessential components of the innate immune system [20], they also function as links to the acquired immune system [4, 21]. This linking role might put these defences at the centre of trade-offs between branches of the immune system.

Quantification and among-species comparison of acquired imunity is methodologically more challenging, since many acquired defences and related assays (e.g., total IgY) are heavily dependent on prior antigenic exposure [27]. Thus, focusing on constant (i.e., genetic) components of acquired immunity should provide a more reliable approach to comparative research. Here, we focused on the structural complexity of the MHC region, which is a key component of acquired immune system in vertebrates. In general, the level of MHC polymorphism is positively related to the diversity of pathogens recognized by the acquired immune system [28]. A higher number of expressed MHC molecules allows an animal to recognize a broader spectrum of antigens (although high MHC variation may also restrict T cell receptor repertoire [29]), and within-individual MHC allelic richness is primarily constrained by the number of duplicated MHC gene copies within the genome. Large variation in MHC gene copy number exists among vertebrates, including birds [30, 31], and comparative studies clearly indicate that this variation is adaptive, correlating with important life history components and ecological traits that are linked to pathogen exposure [30]. In fact, direct comparative evidence of associations between MHC polymorphism (in terms of copy numbers and allelic richness) and pathogen or parasite richness suggests that among-species variation in structural complexity of the MHC region was shaped by historical pathogen-driven selection [32–34]. Thus, variation in the MHC gene copy number should reflect long-term macroevolutionary selection for antigen recognition capacity by the acquired immune system and thereby serve effectively in the analyses of coevolution between acquired immunity and innate immune defences that were previously shown to have a relatively strong genetic component [35–37].

According to the trade-off hypothesis, we predicted that species with more copies of MHC genes would show lower concentrations of natural antibodies and complement. While the costs of development, maintenance, and use of natural antibodies and complement are thought to be relatively low [11], downregulation of these defences has been documented during or after some energetically-demanding periods (e.g., endurance flight [38]), indicating that there is evolutionary potential for innate immunity to have been suppressed among species under limited resources. Also, individuals or species with more diverse MHC repertoires (i.e., larger numbers of gene copies) are thought to suffer greater physiological costs [39], which can promote fitness trade-offs between innate and acquired immunity. Alternatively, under the antigen exposure hypothesis, species could maximize immunological investment overall, producing positive evolutionary associations between MHC complexity and innate immune defences. Finally, since any associations between immune defences could be primarily driven by species ecology and life history (the pace-of-life hypothesis), we accounted for this kind of variation in our analyses.

## Materials and methods

### Data compilation

As indices of the innate immune system, we used titres of haemagglutination and haemolysis, which were quantified following the protocol of Matson et al. [19]. To briefly summarize the assay, haemagglutination and haemolysis were quantified in serially diluted plasma samples that were incubated with exogenous red blood cells. Haemagglutination occurs when the natural antibodies in a (diluted) sample cause the blood cells to clump or agglutinate; haemolysis occurs when complement, interacting with the natural antibodies, causes the cells to lyse.

Independent of the current analyses, a dataset on haemagglutination and haemolysis values was assembled using peer-reviewed studies of birds that cited the original assay protocol of Matson et al. [19]. In assembling that dataset, (sub)studies involving modifications to the assay protocol that could be expected to impact results were excluded. When a study included “standard” (normal incubation periods, healthy control bird, etc.) and “non-standard” conditions (longer or shorted incubation periods, immune challenged birds, etc.), the values under standard conditions were still used. In rare cases where a full study was excluded (e.g., study that used rat red blood cells instead of rabbit red blood cells in a study of house sparrows, *Passer domesticus* [40]), values for the study species were generally available elsewhere. Ultimately, the need for corresponding MHC data (below) acted as a more selective filter than any methodological exclusions (e.g., sky lark *Alauda arvensis* [41] and budgerigar *Melopsittacus undulatus* [42] values under standard and nonstandard assay conditions are available, but MHC data are not). Thus, the current analyses were largely, if not wholly, unaffected by the haemagglutination-haemolysis dataset assembly criteria.

Means or medians (if mean values were not available) for haemagglutination and haemolysis were extracted either directly from the texts and tables or indirectly (using GetData Graph Digitizer) from the figures. In our final dataset (see below), we had a per-species average of 5.1 ± 7.9 [SD] and 6.5 ± 8.9 [SD] independent estimates of haemagglutination and haemolysis, respectively. These estimates were based on an average sample size of 22.2 ± 17.6 [SD] individuals for haemagglutination and 24.3 ± 10.8 [SD] individuals for haemolysis (data and sources provided in Additional file 1). Species-specific values were calculated for each trait as averages across all independent estimates. Our preliminary analyses revealed high intra-specific repeatability of both haemagglutination (R = 0.92, 95% CI 0.86–0.96, P < 0.001) and haemolysis (R = 0.97, 95% CI 0.93–0.99, P < 0.001), indicating that mean values are likely to reliably reflect species-specific activity levels of the corresponding components of the innate immune system (natural antibodies and complement). We also did not find any effect of sample sizes on mean values of haemagglutination (r = − 0.27, P = 0.31) or haemolysis (r = − 0.03, P = 0.89), indicating that our data were not biased by differences in sampling effort.

The MHC gene copy number was used as an index of the acquired immune system. In general, the MHC expansion (an increase in gene copy number via duplication) is a key evolutionary step enhancing within-individual MHC allelic diversity and increasing the spectrum of antigens recognized by the acquired immune system. Although a positive association between gene copy numbers and individual MHC diversity can be obscured in short evolutionary scales by demographic processes (e.g. by genetic bottlenecks producing high levels of homozygosity across duplicated genes), an overall variation in MHC gene copy numbers is expected to primarily reflect long-term macroevolutionary selection shaping antigen recognition capacity of the acquired immune system (constrained by the mechanisms selecting against the high number of copies in the genome) [43]. For the purpose of this study, we primarily relied on a previously published comparative dataset that was used to reconstruct evolution of avian MHC duplication patterns [30]. The dataset has been updated with information from several more recent publications [31, 44–48]. Information on copy number was compiled for both MHC class I and class II genes (henceforth referred to as MHC-I and MHC-II), which are primarily involved in recognition of intra- and extra-cellular antigens, respectively (although extra-cellular antigens may also be cross-presented by MHC-I [49]). The estimates of MHC copy number were based exclusively on the putatively functional genes; any gene copies showing signatures of pseudogenization (e.g., stop codons or frame-shift mutations) were excluded. We exclusively focused on classical MHC genes. Non-classical MHC genes, which can show low polymorphism, low or tissue-specific expression, or altered functionality [50], may be present in some avian species. We excluded all identified non-classical genes (e.g. MHC-Y genes in Phasianidae [51]) from our copy number estimates. MHC-II copy number estimates were based on loci coding for a beta subunit, which are generally more polymorphic and show stronger signature of positive selection than those coding for an alpha subunit [52].

Since MHC genes are often highly duplicated, locus-specific genotyping is not possible in most non-model species and copy numbers are estimated using a variety of methods. In our final dataset (see below), most estimates were obtained directly from third-generation genome assemblies (30.2%) or indirectly, calculated as the maximum number of MHC alleles per individual retrieved from next-generation sequencing (NGS; 41.9%) or cloning (20.9%). A minority of estimates (7.0%) were obtained using other methods. We found no significant differences in the estimates of mean copy number among the genotyping methods (ANOVA: P > 0.05 for MHC-I and MHC-II). Likewise, we found that copy number estimates did not covary with genotyping effort (log number of genotyped individuals) for NGS techniques (P > 0.05). Although we concluded that our MHC data were unlikely to show any major methodological biases, we also controlled for methodological variation in the models that we ran separately for each MHC class (family-level analyses, see Phylogenetically-informed analyses section for details). For this purpose, we extracted the dominant method, i.e., either the most prevalent method (as was the case for 18.75% of families) or the only method (as was the case for the remaining 81.25% of families) used to estimate MHC-I or MHC-II gene copy number among species representing each family. In species where data on copy number were missing for a single MHC class (4 and 7 species for MHC-I and MHC-II, respectively), copy number for the second class was estimated using information from closely related species (from the same genus), if possible. We expected this approach to be methodologically reliable, as copy number estimates showed relatively high repeatability within genera (23 estimates from five genera; R = 0.78, P = 0.015). However, to further assess reliability of this approach we re-ran the analyses using family-level means of our immune indices, which were calculated directly from our data with no extrapolations (data in Additional file 1). All reported intra-specific repeatabilities were calculated as the two-way intra-class correlation coefficients in *irrNA* package [53] developed for R statistical environment (R Foundation for Statistical Computing, Vienna, Austria).

### Final dataset: innate and acquired immune traits

**Fig. 1:**
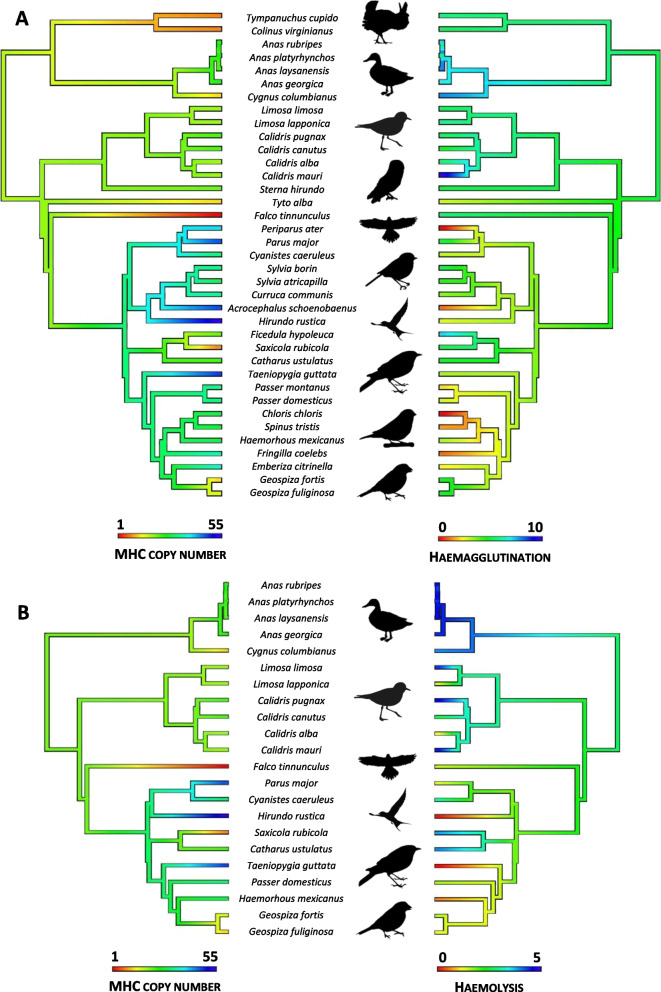
Phylogenetic distribution of acquired (total MHC copy number) versus innate immune defences (**A** haemagglutination, **B** haemolysis) in birds

In total, full data on haemagglutination and MHC copy number were available for 37 species (Fig. 1A), while data on haemolysis and MHC were available for 22 species (Fig. 1B). Although the original data on haemagglutination, haemolysis, and the MHC were much more extensive (included many more species), the datasets did not match well in terms of species composition. Despite our modest sample sizes, our final dataset had relatively broad phylogenetic coverage, including 18 extant bird families from six orders (10 families from four orders for haemolysis). Within this dataset, haemagglutination ranged from 0.0 to 9.9 (mean 4.6 ± 2.4 [SD]), while haemolysis ranged from 0.02 to 4.8 (mean 2.7 ± 1.7 [SD]). As both traits positively correlated (raw values: r = 0.65, P = 0.001) and, thus, may not have been fully independent (despite being based on different immunological mechanisms), we also calculated a combined haemagglutination-haemolysis score. For this purpose, we z-transformed both variables and either calculated means across the two scores (if both values were available) or used haemagglutination score (if haemolysis score was missing). The copy number of MHC-I and MHC-II genes ranged from 1 to 33 (mean 6.9 ± 7.3 [SD]) and from 1 to 43 (mean 5.9 ± 8.5 [SD]), respectively, and both traits also positively correlated (non-extrapolated raw values: r = 0.51, P = 0.042). Because the measured innate immune defences can act against both intra- and extra-cellular pathogens [18] and because of the possibility of cross-presentation by MHC molecules [42], we primarily aimed to analyse MHC-I and MHC-II data jointly. For this purpose, we summed both estimates of copy numbers for each species (2 to 55, mean 12.8 ± 11.6 [SD]) and used log-transformed total values (due to strong right-skewness: 2.11) in our analyses.

### Life history and biogeographical data

Since the pace of life and ecology of species have been linked theoretically and empirically to immune defences [9, 11, 30] and may shape the relative investment in either axis of the immune system [10], we also tested if evolutionary associations between the innate and acquired immune defences in birds are governed by the key ecological and life history traits. First, we compiled data on body mass, as due to size-specific variation in metabolic rate, body mass is considered one of the key determinants of fast-low life history continuum (pace of life) [54]. Second, we compiled information on longevity and calculated residual lifespan (residuals of phylogenetically-informed regression between maximum lifespan and body mass) for each species. Finally, we also collected information on the extent of migration (three categories: resident, short-distance, and long-distance) and the breeding latitude, since pathogen-driven selection on the MHC is higher in migratory species and may vary biogeographically [30, 55]. Details on data sources and processing are provided in Additional file 2 (Supplementary Methods).

### Phylogenetically-informed analyses

We used a phylogenetically-informed Bayesian mixed model framework (as implemented in the *MCMCglmm* R package [56]) to test for associations between MHC copy number and haemagglutination and haemolysis titres. Due to differences in sample size, we separately analysed haemagglutination and haemolysis. First, we used a single-predictor approach at both the species and family level (i.e., species- and family-specific means used as the unit of analysis). The total MHC copy number was entered as the response variable, and haemagglutination and haemolysis scores were each entered as the explanatory variable in two separate models. To assess if any significant relationships were primarily driven by copy number variation within either MHC-I or MHC-II region, we re-ran family-level analyses (non-extrapolated data) separately for both MHC classes. To control for methodological variation, we also re-ran these models with methods used to estimate MHC-I and MHC-II gene copy number included as the moderator. To assess effect sizes, we re-ran the species-level single-predictor models with z-transformed values of immune traits and compared the regression slopes using analysis of covariance. Second, we ran species-level multi-predictor models with the intention of controlling for variation in ecology and life history. In these models MHC copy number was also used as the response variable, while log body mass, residual lifespan, and breeding latitude were added as covariates and the extent of migration was added as a fixed factor in addition to either haemagglutination or haemolysis score. Finally, both single- and multiple-predictor models were re-run using the combined haemagglutination-haemolysis score as the explanatory variable, in place of haemagglutination or haemolysis.

All the models were run with two independent MCMC chains with 200 000 iterations, a thinning value of 100, and a burn-in period of 50 000, resulting in 1 500 expected samples per chain. The chains effectively converged in all models, as assessed with the effective sample sizes of 1473.8 ± 3.3 [SE] and the multivariate potential scale reduction values < 1.05 [57]. Each model was run for the Gaussian distribution of the response variable and under uninformative priors (variance set to 1 and belief parameter set to 0.002 for both R and G variance structures). Phylogeny was included as a random effect in all the models. Information on phylogenetic relationships among species and families was based on the complete avian time-calibrated phylogeny [58] with a backbone topology provided by Ericson et al. [59], as retrieved from the BirdTree webserver (http://www.birdtree.org). To account for phylogenetic uncertainty, all coefficient estimates were averaged across 100 randomly selected phylogenies using the *multree* R package [60]. For each model, we calculated the marginal R^2^ (variance explained by the fixed effects only) and the conditional R^2^ (variance explained by fixed and random effects). The difference between the two R^2^ estimates indicates the proportion of variation explained by phylogeny (random factor). Immune defences (MHC copy number, haemagglutination, and haemolysis) were mapped on the phylogeny using the *phytools* R package [61]. All values are reported as means ± SE.

## Results

**Fig. 2:**
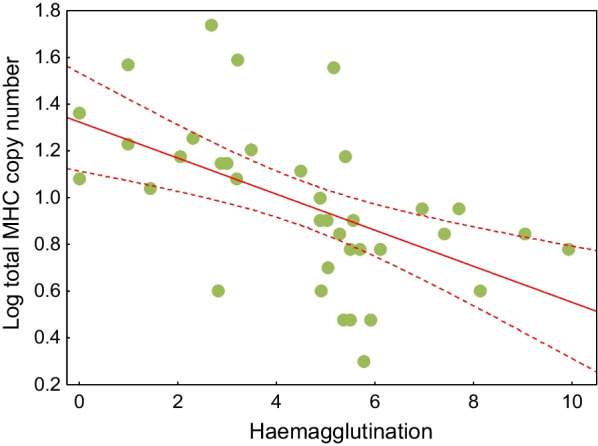
Species-level association of the total MHC gene copy number (acquired immunity) with haemagglutination (innate immunity) in birds. Regression lines with 95% confidence intervals are shown

**Fig. 3:**
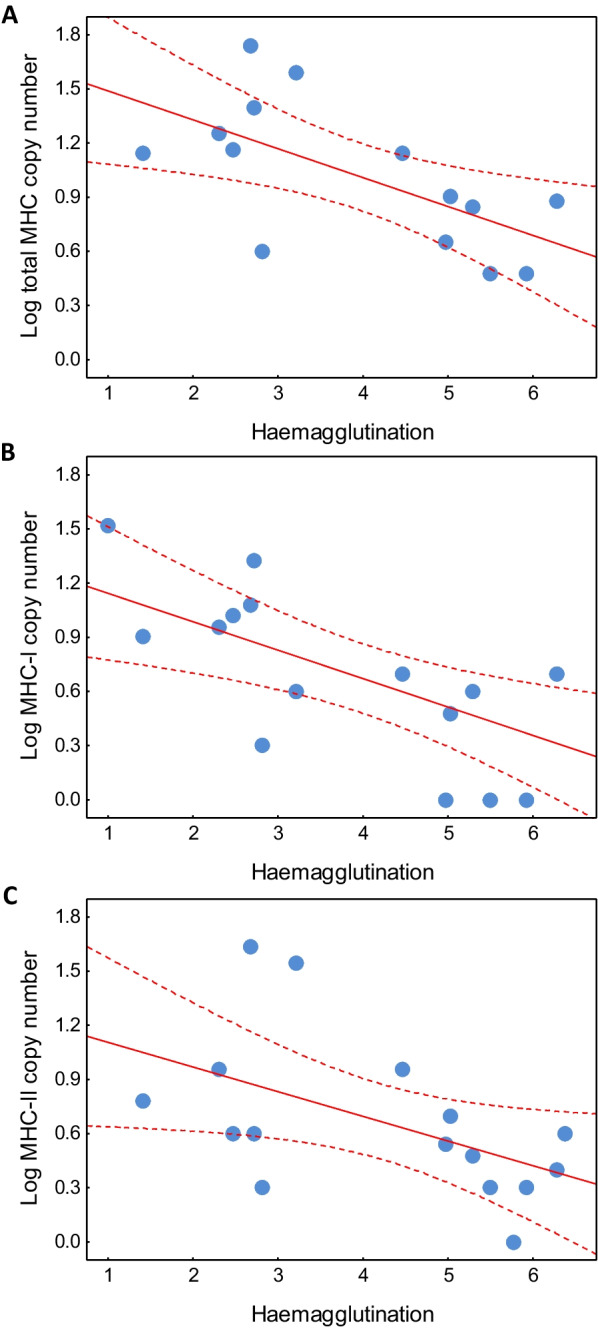
Family-level associations of MHC gene copy number (acquired immunity) with haemagglutination (innate immunity) in birds, as assessed across the total MHC (**A**), MHC class I (**B**), and MHC class II (**C**) copy number. Regression lines with 95% confidence intervals are shown

We found a negative association between the total MHC gene copy number and haemagglutination in birds, indicating MHC expansion (higher copy number) in species with lower innate immune defences (lower natural antibody concentrations), as revealed by the single-predictor model at the species level (β = − 0.049, 95% CL − 0.089 to − 0.013, P = 0.011; Fig. 2). The species-level association remained significant when tested using the multi-predictor model that controlled for variation in ecology and life history (Table 1). In this analysis, body mass was the only other significant predictor of MHC copy number (also negative; Table 1). A negative association between the MHC gene copy number and haemagglutination was also retained at the family level (β = − 0.151, 95% CL − 0.280 to − 0.023, P = 0.021; Fig. 3A) and it was apparent for both MHC-I (β = − 0.157, 95% CL − 0.273 to − 0.056, P = 0.007; Fig. 3B) and MHC-II (β = − 0.129, 95% CL − 0.264 to − 0.003, P = 0.050; Fig. 3C), when analysed separately. The latter two associations remained significant after accounting for the methods used to estimate the MHC gene copy number (P = 0.043 for MHC-I; P = 0.025 for MHC-II). The proportion of variance explained by fixed effects (marginal R^2^) in the single-predictor species- and family-level models was 0.152 and 0.432, respectively, while in the multi-predictor species-level model it was 0.479. The proportion of variance explained by fixed and random effects (conditional R^2^) ranged from 0.954 to 0.991, depending on the model. The substantially higher conditional R^2^ values indicated that phylogeny explained a major part of the variation in the data.

**Table 1:** The results of Bayesian phylogenetic mixed models testing associations between MHC gene copy number (acquired immunity) and haemagglutination (innate immunity) in birds

Predictor	Estimate	Lower 95% CL	Upper 95% CL	p
Intercept	**1.395**	**0.975**	**1.811**	** < 0.001**
Haemagglutination	**− 0.042**	**− 0.080**	**− 0.003**	**0.033**
Log body mass	**− 0.237**	**− 0.407**	**− 0.065**	**0.007**
Residual lifespan	0.258	**− **0.170	0.687	0.241
Breeding latitude	0.007	0.000	0.013	0.057
Extent of migration	**− **0.113	**− **0.249	0.020	0.100

Coefficient estimates with corresponding 95% credibility limits (CL) and p-value are shown for each predictor; all values were averaged across 100 different phylogenies. Significant coefficients are marked in bold

In contrast to haemagglutination, we found no support for a significant association between MHC copy number and haemolysis in the single-predictor analyses (species level: β = − 0.074, 95% CL − 0.171–0.022, P = 0.132, marginal R^2^ = 0.128, conditional R^2^ = 0.938; family level: β = − 0.136, 95% CL − 0.365–0.093, P = 0.247, marginal R^2^ = 0.004, conditional R^2^ = 0.990; Additional file 2: Fig. S1) or in the multi-predictor model (Additional file 2: Table S1). However, the regression slopes from species-level single-predictor models using z-transformed immune trait values did not differ significantly (β_haemolysis_ = − 0.381 and β_haemagglutination_ = − 0.323; P = 0.92; Additional file 2: Table S2). Also, there was a significant negative association between the total MHC gene copy number and the combined haemagglutination-haemolysis score at the species level, as revealed by both single-predictor model (β = − 0.150, 95% CI − 0.255 to − 0.046, P = 0.005) and multi-predictor model (Additional file 2: Table S3).

## Discussion

Our comparative analyses provided support for a negative association between the expression of the innate and acquired immune traits in birds. We found that species that evolved more complex MHC architecture with extensive tandems of duplicated genes (meaning a broader spectrum of antigens being recognized by the acquired immune system) had lower concentrations of innate (germline encoded) natural antibodies, which were measured via haemagglutination. Importantly, we also found that this association was not primarily driven by life history variation among species. No significant association was found between MHC architecture and complement, which was measured via haemolysis. Nevertheless, statistical analyses of haemolysis and haemagglutination resulted in similar negative slopes, and both innate indices explained a similar amount of variation in MHC copy number (13% vs. 15% in the species-level single-predictor models). Thus, the lack of statistical significance in the haemolysis analysis could be due to the smaller sample size (only 22 species) and corresponding lower statistical power. Alternatively, a systematic underrepresentation of low lysis values in published studies could have undermined this relationship.

In general, acquired immune defences are considered more evolutionarily derived than innate immune defences. The evolution of immune defences in metazoans proceeded from non-specific phagocytic cells with conserved receptors towards higher specificity, antibody maturation, and immunological memory [1]. Similarly, within different vertebrate lineages, evolution has driven increasingly complex acquired defence mechanisms. The evolutionary history of the MHC region in birds exemplifies these processes. The most basal bird lineages (Palaeognathae and Galliformes) have simple MHC architecture with tightly clustered MHC-I and MHC-II genes and few duplications [62, 63]. For instance, most galliforms have maximally three MHC genes per class [30, 62], and the chicken *Gallus gallus* has only one dominantly expressed gene per class (a so-called minimal essential MHC [51]). Relatively simple MHC architecture has been conserved throughout most non-passerine lineages (with some exceptions, e.g., 12 functional MHC-I genes reported in the carmine bee-eater *Merops nubicus* [31]), and extensive MHC expansion did not occur until the relatively recent (Cenozoic) radiation of passerines (Passeriformes). Dozens of duplicated MHC genes have been found in some passerine genomes, and some passerine clades show an extraordinary copy number variation (e.g., the nearly 200 MHC-II genes found in manakins Pipridae [31]). Recent genomic analyses suggest that this large inter-specific variation in the MHC copy number may have been primarily caused by tandem duplications of classical MHC genes often located outside the core MHC region [64]. In contrast to MHC architecture, reconstructions of evolutionary histories of innate immune defences at broad phylogenetic scales are limited in birds (with the notable exception of toll-like receptors, TLRs [65]). Thus, we have little information on the phylogenetic distribution of levels of natural antibodies and complement. Previous comparative research revealed only a weak phylogenetic signal in these defences, but those analyses were restricted almost exclusively to Neotropical passerines [9], and further evaluation across the avian tree of life is needed. Nevertheless, our results show a negative relationship between MHC gene copy number and natural antibodies and suggest an evolutionary trade-off between two avian immunological subsystems: more developed acquired immune defence (i.e., more complex MHC architecture) in more derived bird lineages (e.g., passerines) is accompanied by constrained innate immune defence. At the same time, it needs to be explicitly stated that our analyses are of correlative nature and, thus, they do not allow inferences on causality, including direct causal inferences on functional trade-offs between our immune traits. Also, our inferences were based on the analysis of a single component of the innate and acquired immune system and we lack quantitative information on whether and how the expression of these traits (e.g. haemagglutination) correlates with other immune variables (e.g. other components of cellular immunity). It may well be possible that some immune traits are more prone to be involved in evolutionary (e.g. resource allocation) trade-offs than the others and the global picture of evolutionary inter-relationships between different pathways of the avian immune system is likely to be far more complex than presented here.

Our multi-predictor models do not support either the pace-of-life hypothesis or the pathogen exposure hypothesis, both of which are commonly invoked to explain immunological variation [9–12, 15]. We found that the negative association between MHC copy number and haemagglutination retained significance after controlling for basic life history components and for ecological traits commonly linked to pathogen exposure (e.g., extent of migration). Nevertheless, we acknowledge that both innate and acquired immune defences almost certainly respond evolutionarily to pathogen-driven selection and are likely optimized within a life history context. For example, inter-specific analyses in birds revealed that levels of both natural antibodies and complement varied with basic reproductive traits, such as developmental period and clutch size [9, 66]. Likewise, MHC copy number was found to be correlated with avian body mass and lifespan [30]. Significant variation along axes of life history has also been described for other avian immune traits, including acquired T-cell mediated immunity [67] and leukocyte concentrations [66, 68]. Similar associations have been reported in vertebrates other than birds [14, 69]. Ultimately, this current understanding of evolutionary variation in immune defences within a framework of life history is not at odds with the results we report here. Regardless of variation in particular types of immune defences, the trade-off between them may constitute a general pattern, which itself is not connected to life history.

Ideas related to the evolution of trade-offs between the innate and acquired immunity have arisen from empirical research in different study systems. For example, an evolutionary transition from high MHC variation (tens of allelic variants per species) in the basal falcon lineage (kestrels) towards extremely low polymorphism of these genes (1–5 allelic variants per species) in more derived falcon lineages has been reported [70]. This impoverishment of MHC repertoire was not accompanied by reduction in genome-wide heterozygosity and has not constrained the ecological radiation of falcons. Thus, it has been proposed that strong innate immune defences may have limited the need for the development of strong acquired immune defences in more derived falcon lineages, although this hypothesis has not been explicitly tested [70]. Beyond falcons, empirical support for this hypothesis has been found in a comparison of wild felid species. Low acquired immunity (specifically low MHC diversity) in the cheetah *Acinonyx jubatus* was accompanied by elevated innate immunity, while the opposite pattern was found in the leopard *Panthera pardus* (higher MHC diversity and weaker innate immunity [71]). More evidence for the evolution of immunological trade-offs comes from genomic research on the Atlantic cod *Gadus morhua*. A striking expansion of innate pathogen-recognition receptors, especially nucleic-acid-detecting TLRs, was accompanied by a complete loss of acquired immunity (MHC-II) genes in this species [72], suggesting that cods rely more heavily on the innate defences (TLR pathway) to recognize bacterial pathogens compared to other teleost fish [72]. Similarly, research on three-spined sticklebacks *Gasterosteus aculeatus* that were artificially selected for low innate immunity also pointed to a trade-off mechanism: they exhibited increased (by ca. 50%) expression of MHC-II compared to individuals with stronger innate immunity [73]. Intra-specific analyses linking MHC variation with innate immune traits in birds were scarce and largely inconclusive [74, 75], suggesting that individual optimization of immune responses may not always match with macroevolutionary adaptations.

A negative relationship between the innate and acquired branches of the immune system, as we report here for birds, could reflect immune compensation driven by trade-offs in resource allocation. It is widely accepted that immune systems incur costs and that immunological investments come at the expense of other vital processes [5, 6]. Thus, animals are expected to optimize (rather than maximize) their immune system and fine tune their overall immunological strategy with respect to life history, ecology, and pathogen exposure [10]. For example, scavenging birds, whose feeding strategy involves exposure to abundant and diverse pathogens and other microbes, are hypothesized to have experienced strong selection pressures for innate immunity [76]. Consistent with this hypothesis, scavengers had a lower proportion of lymphocytes than phagocytic leukocytes, suggesting strong front line immune defences, which could potentially reduce the need for mounting lymphocyte-dependent acquired immune responses [76]. Since the ultimate goal of the immune system is to protect an individual (and thereby maximize its fitness) and different types of immune defences can at least partially compensate for each other, trade-offs resulting in different strategies are expected to prevail, while concurrent across-the-board immunological investments are not. Of course, the possibility exists that higher levels of a particular element of the immune system might counterintuitively lower the overall (e.g., metabolic) costs associated with certain immune functions. However, in such a case, a negative relationship between immune defences, like the one we observed here, would not be expected. In the case of competing costs, simultaneously maximizing all types of immune defences may be too expensive and not optimal for most, but perhaps not all, species. In fact, some species may invest heavily to maintain defences associated with both branches, especially under strong pathogen-driven selection. For example, among five studied species of shorebirds (Charadrii), the ruddy turnstone *Arenaria interpres* exhibited the highest levels of both innate and acquired defences, which could reflect exposure to diverse pathogen communities [77].

In conclusion, our study provides comparative evidence for the evolutionary co-optimization of the innate and acquired branches of the avian immune system. Our results support established theoretical frameworks in biomedical and ecological immunology [7], which have so far received relatively little attention from evolutionary biologists and, thus, lacked empirical support across diverse species. Overall our findings offer new insights into evolutionary compromises within the avian immune system, while simultaneously highlighting the need to test similar hypotheses within and among other vertebrate lineages with diverse life histories. Ideally, future investigations will continue to employ both functional (here, haemagglutination and haemolysis) and genomic (here, MHC architecture) indices that reflect different immunological branches and subsystems. For example, tissue graft rejection speed could be a functional test of acquired defences, whereas the polymorphism of pattern-recognition receptor genes could serve as a genomic index of innate defences. However, the broad application of many immunological tests and techniques outside of model species (and thus beyond intra-specific investigations) presents major logistical hurdles [19]. Ultimately, conducting the type of phylogenetically diverse comparative analyses we call for here necessitates careful standardization of immunological assays that are practicable for use in a wide range of wild animals in field settings. We though acknowledge that even under standardized protocols, high seasonality or immune remodelling across individual lifetimes might generate shifts in relative acquired-innate immune investment either cyclically or directionally, respectively. This might be an important consideration for future sampling regimes that aim to assess evolutionary trade-offs, which could be overwhelmed by within-individual (potentially adaptive) variation.

### Supplementary Information

**Additional file 1.** Raw data used in the analyses.

**Additional file 2.** Supplementary methods and supplementary results of Bayesian phylogenetic mixed models testing associations between innate and acquired immune defences in birds (Tables S1–S3 and Fig. S1).

## Data Availability

All data used in the analyses are provided in the Additional file 1.
