# Supplementary material for: Evolutionary trade-off between innate and acquired immune defences in birds
Source: Front Zool. 2023 Sep 8;20:32. doi: 10.1186/s12983-023-00511-1 (PMC10486109; doi:10.1186/s12983-023-00511-1)
Supplement: Supplementary file 2 — Additional file 2. Supplementary methods and supplementary results of Bayesian phylogenetic mixed models testing associations between innate and acquired immune defences in birds (Tables S1–S3 and Fig. S1). [file 12983_2023_511_MOESM2_ESM.docx]

**Additional File 2**

**Evolutionary trade-off between innate and acquired immune defences in birds**

**Piotr Minias^1^*, Wei-Xuan V.-H. Peng^2^ and Kevin D. Matson^2^**

^1^ Department of Biodiversity Studies and Bioeducation, Faculty of Biology and Environmental Protection, University of Łódź, Banacha 1/3, 90-237 Łódź, Poland

^2^ Wildlife Ecology and Conservation Group, Wageningen University & Research, Droevendaalsesteeg 3a, 6708PB Wageningen, Netherlands

*Correspondence:

Piotr Minias

pminias@op.pl

**Supplementary Methods**

**Life-history and biogeographical data**

For each species, we compiled data on body mass, maximum lifespan, migration distance, and breeding latitude. Data on body mass were compiled from standard references (del Hoyo et al. 1992-2011) as averages across both sexes, while maximum lifespan estimates were retrieved from the AnAge database (de Magalhães and Costa 2009) integrated within the web portal of the Human Ageing Genomic Resources (Tacutu et al. 2013). Since vertebrate lifespan shows an allometric association with body size (larger species live longer; Speakman 2005) and longevity records may depend on sampling effort (probability of recording an extremely old individual increases with sample size) or source of data (lifespan is longer in captivity than in the wild) (Møller 2007), we aimed to remove background variation prior to the analyses. For this purpose, we used a phylogenetically-informed framework (phylogenetic generalized linear models, PGLS; Martins and Hansen 1997), since ignoring phylogeny during preliminary transformations of interspecific data can elevate variance and type I error (Revell 2009). Information on phylogenetic relationships between species was based on the complete avian time-calibrated phylogeny by Jetz et al. (2012). We downloaded a random sample of one thousand trees (backbone topology by Ericson et al. 2006) from the BirdTree database (Jetz et al. 2012) and then extracted a single consensus tree in Geneious v10.0.5 software (Biomatters Ltd., Auckland, New Zealand). All the models were run using the Brownian Motion model of evolution in the *ape* R package (Paradis et al. 2004). We found no evidence for associations of log lifespan with sampling effort (F_3,32_ = 0.65, P = 0.59) or source of data (F_1,34_ = 0.09, P = 0.77), but there was a positive linear association with log body mass (β = 0.101 ± 0.044, F_1,35_ = 2.29, P = 0.028). Thus, we extracted residuals from the log-log PGLS regression between lifespan and body mass and used them in the analyses (henceforth referred to as residual lifespan). Breeding latitude and migration distance for each species were quantified using distribution maps (shape files) downloaded from BirdLife International website (BirdLife International & Handbook of Birds of the World 2016) and *gCentroid* function in the *rgeos* R package (Bivand et al. 2017), following methodology proposed by Vincze (2016). Briefly, coordinates of breeding range centroids were calculated using breeding and resident spatial polygons, while coordinates of wintering range centroids were calculated using wintering and resident spatial polygons. Migration distance was calculated as the geographic distance between both (breeding and wintering) centroids using a custom R function developed by Vágási et al. (2016). Since migration distance within our dataset was highly zero-inflated, we could not use it as a linear covariate in the analyses. Instead, we categorized species into three categories based on extent of migration (residents species: 0 km; short-distance migrants: 0-2000 km; long-distance migrants: > 2000 km) and used this categorization in our analyses. Centroids of breeding range latitudes were used as breeding locations for species (referred to as breeding latitude in the models).

**References**

BirdLife International and Handbook of Birds of the World. 2016. Bird species distribution maps of the world, ver. 6.0. Available at http://datazone.birdlife.org/species/requestdis

Bivand R, Rundel C, Pebesma E, Stuetz R, Hufthammer KO. 2017. Rgeos: Interface to Geometry Engine – Open Source (GEOS). R package ver. 0.3-23. Available at https://CRAN.R-project.org/package=rgeos

de Magalhães JP, Costa J. 2009. A database of vertebrate longevity records and their relation to other life-history traits. J Evol Biol. 22:1770–1774.

del Hoyo J, Elliott A, Sargatal J. 1992-2011. Handbook of the birds of the world (Vol. 1–16). Barcelona, Spain: Lynx Edicions.

Ericson PGP, Anderson CL, Britton T, Elzanowski A, Johansson US, Källersjö M, Ohlson JI, Parsons TJ, Zuccon D, Mayr G. 2006. Diversification of Neoaves: integration of molecular sequence data and fossils. Biol Lett. 2:543–547.

Jetz W, Thomas GH, Joy JB, Hartmann K, Mooers AO. 2012. The global diversity of birds in space and time. Nature. 491:444-448.

Martins EP, Hansen TF 1997. Phylogenies and the comparative method: A general approach to incorporating phylogenetic information into the analysis of interspecific data. Am Nat. 149:646–667.

Møller AP. 2007. Senescence in relation to latitude and migration in birds. J Evol Biol. 20:750-757.

Paradis E, Claude J, Strimmer K. 2004. APE: Analyses of phylogenetics and evolution in R language. Bioinformatics. 20:289–290.

Revell LJ. 2009. Size-correction and principal components for interspecific comparative studies. Evolution. 63:3258–3268.

Speakman JR. 2005. Body size, energy metabolism and lifespan. J Exp Biol. 208:1717-1730.

Tacutu R, Craig T, Budovsky A, Wuttke D, Lehmann G, Taranukha D, Costa J, Fraifeld VE, de Magalhães JP. 2013. Human ageing genomic resources: integrated databases and tools for the biology and genetics of ageing. Nucl Acids Res. 41:D1027–D1033.

Vágási CI, Pap PL, Vincze O, Osváth G, Erritzøe J, Møller AP. 2016. Morphological adaptations to migration in birds. Evol Biol. 43:48–59.

Vincze O. 2016. Light enough to travel or wise enough to stay? Brain size evolution and migratory behaviour in birds. Evolution. 70:2123–2133.

**Table S1** The results of Bayesian phylogenetic mixed models testing associations between MHC gene copy number (acquired immunity) and haemolysis (innate immunity) in birds. Coefficient estimates and corresponding 95% credibility limits (CL) are shown for each predictor; all values were averaged across 100 different phylogenies. Significant coefficients are marked in bold.

| Predictor | Estimate | Lower 95% CL | Upper 95% CL | p |
| --- | --- | --- | --- | --- |
| **Intercept** | **1.303** | **0.638** | **1.982** | **<0.001** |
| Haemolysis | -0.041 | -0.155 | 0.073 | 0.493 |
| Log body mass | -0.207 | -0.538 | 0.116 | 0.216 |
| Residual lifespan | 0.415 | -0.615 | 1.438 | 0.437 |
| Breeding latitude | 0.005 | -0.008 | 0.018 | 0.468 |
| Extent of migration | -0.095 | -0.346 | 0.114 | 0.424 |

**Table S2** The results of Bayesian phylogenetic mixed models testing associations between MHC gene copy number (acquired immunity) and z-transformed values of innate immune indices (haemagglutination and haemolysis) in birds. Coefficient estimates and corresponding 95% credibility limits (CL) are shown for each predictor; all values were averaged across 100 different phylogenies. Significant coefficients are marked in bold

| Dependent variable | Predictor | Estimate | Lower 95% CL | Upper 95% CL | p |
| --- | --- | --- | --- | --- | --- |
| Haemagglutination (HA) | **Intercept** | **0.912** | **0.754** | **1.091** | **<0.001** |
|  | **Z-transformed HA** | **-0.117** | **-0.206** | **-0.022** | **0.011** |
| Haemolysis (HL) | **Intercept** | **-0.907** | **0.624** | **1.172** | **<0.001** |
|  | Z-transformed HL | -0.124 | -0.302 | 0.029 | 0.129 |

**Table S3** The results of Bayesian phylogenetic mixed models testing associations between MHC gene copy number (acquired immunity) and combined haemagglutination-haemolysis scores (innate immunity) in birds. Coefficient estimates and corresponding 95% credibility limits (CL) are shown for each predictor; all values were averaged across 100 different phylogenies. Significant coefficients are marked in bold.

| Predictor | Estimate | Lower 95% CL | Upper 95% CL | p |
| --- | --- | --- | --- | --- |
| **Intercept** | **1.161** | **0.751** | **1.568** | **<0.001** |
| **Haemagglutination-haemolysis** | **-0.109** | **-0.215** | **-0.003** | **0.043** |
| **Log body mass** | **-0.228** | **-0.401** | **-0.053** | **0.010** |
| Residual lifespan | 0.006 | -0.001 | 0.013 | 0.071 |
| Breeding latitude | -0.093 | -0.229 | 0.038 | 0.175 |
| Extent of migration | 0.270 | -0.158 | 0.702 | 0.221 |

**Fig. S1** Non-significant species-level (A) and family-level (B) associations of the total MHC gene copy number (acquired immunity) with haemolysis (innate immunity) in birds.

**A**

**B**
